# Survival outcomes based on systemic agent used concurrently with radiation in human-papillomavirus associated oropharyngeal cancer

**DOI:** 10.18632/oncotarget.20197

**Published:** 2017-08-10

**Authors:** Vikas Mehta, Tara Moore-Medlin, Jose M. Flores, Xiaohui Ma, Oleksandr Ekshyyan, Cherie-Ann O. Nathan

**Affiliations:** ^1^ Departments of Otolaryngology-Head and Neck Surgery, Louisiana State University Health and Feist-Weiller Cancer Center, Shreveport, LA, USA; ^2^ Johns Hopkins Bloomberg School of Public Health, Baltimore, MD, USA

**Keywords:** oropharyngeal carcinoma, human papillomavirus, cetuximab, cisplatin, survival outcomes

## Abstract

**Purpose:**

To investigate survival outcomes of patients treated with concurrent cetuximab and radiotherapy for primary management of both HPV positive and negative OPSCC, and compare the results to traditional platinum-based therapy. We hypothesize that the use of cetuximab in the HPV positive OPSCC patients will result in inferior survival based on tumor biological differences.

**Study design:**

A single institution retrospective analysis of 304 patients. The primary outcomes of interest were 1) overall survival and 2) relapse free survival. Pearson Chi-square tests were used to compare proportions between subgroups. One-way analysis of variance was used to compare the continuous variable age between subgroups. Kaplan–Meier method was used to produce survival curves, and comparisons between survival curves were made using the log-rank test. The survival functions comparing subgroups of chemotherapy were analyzed using semi-parametric (i.e. Cox proportional hazards models) and fully parametric regression with Weibull distributions. Multivariable models were adjusted for age at diagnosis, gender, race, chemotherapy, radiotherapy, and cancer stage.

**Results:**

In the multivariable analysis, the hazard ratio for cetuximab compared to cisplatin or carboplatin/paclitaxel was HR=0.77[95% CI = 0.67, 0.90] in the HPV - group, suggesting more favorable outcomes for the patients on cetuximab in this group. However, in the HPV + cohort, the hazard ratio was 1.88 [95% CI = 1.42, 2.50] for those patients treated with cetuximab vs platinum-based therapy.

**Conclusions:**

Our data suggest that cetuximab may have inferior outcomes in HPV-associated OPSCC compared to traditional platinum-based therapy.

## INTRODUCTION

Since the discovery of the causal relationship between human papillomavirus (HPV) and oropharyngeal squamous cell carcinoma (OPSCC), there has been a paradigm shift in the field of head and neck oncology. Indeed, what is increasingly evident is that HPV-associated OPSCCA is a completely distinct disease process from the classic variant of head and neck squamous cell carcinoma (HNSCC). This has been shown both on the macro level in terms of epidemiology,[1] presentation,[2] demographics,[3] and prognosis,[4] and on the molecular level with HPV-associated OPSCC containing fewer, reversible and unique pathway alterations.[5] Due to these differences, alternate staging and therapeutic regimens are being proposed to more appropriately manage the HPV + OPSCC patients, and avoid subjecting them to excessive or ineffective treatment.[6] With the improved prognosis and response to treatment, de-escalation using alternative chemotherapeutics with less morbidity than platinum-based therapy is a strategy that is being actively investigated (ECOG 1308, RTOG 1016 and De-ESCALaTE HPV trials).

Bonner et al first introduced cetuximab, an epidermal growth factor receptor (EGFR) monoclonal antibody, in conjunction with radiotherapy for management of HNSCC in 2006 after the publication of their randomized controlled trial demonstrating improved efficacy of concurrent treatment over radiotherapy alone, with no statistically significant increase in morbidity.[7] Since the results of this trial, cetuximab has been incorporated into the armamentarium for treating patients in both the primary and recurrent setting.[8-10] Due to the cost and a lack of trial data directly comparing cetuximab to platinum-based therapy, cetuximab is often administered in the primary setting to those patients where platinum therapy is contraindicated or the patient is subjectively felt to be too frail to tolerate the morbidity of traditional chemoradiation.

Due to the improved side-effect profile, cetuximab is currently being explored as a possible way of de-escalating treatment for HPV associated OPSCC. However, when looking at HPV associated OPSCC on a genomic level, this does not appear to be prudent from a mechanistic standpoint. HPV infection and EGFR gene copy number gain have been shown to be mutually exclusive events in OPSCC[11-13] suggesting there is not a biological rationale for using anti-EGFR therapy for HPV(+) OPSCC. Our goal was to investigate our experience with concurrent cetuximab and radiotherapy for primary management of both HPV positive and negative OPSCC, and compare the results to traditional platinum-based therapy. We hypothesize that the use of cetuximab in the HPV positive OPSCC patients will result in inferior survival.

## RESULTS

### Description of population

Table 1 shows the baseline characteristics of the OPSCC patient cohort separated by HPV(-) (n=104) and HPV(+) (n=172) status. The majority of patients were male in both groups; 75% in HPV(-) and 89.5% in HPV(+) groups. Also, the majority of patients in both cohorts were current or former smokers: 90.4% of HPV(-) and 72.1% of HPV(+). HPV(-) patients had a higher median pack-years of smoking exposure compared to HPV(+) patients (30 vs. 20 pack-years). In each group ∼71% of the patients received chemotherapy with radiotherapy as primary concomitant treatment, while 5.8% of HPV(-) and 9.3% of HPV(+) participants received adjuvant chemotherapy. Table 2 shows the cohort characteristics by chemotherapy treatment modality. Of note, there was a slightly higher prevalence of stage 4 disease in the cetuximab group than in those cohorts where cisplatin was utilized. There were no other significant differences between the treatment arms including HPV status, age, race, gender, and substance abuse.

**Table 1:** Clinical characteristics based on HPV serology

	HPV ((-))	HPV ((+))
Sample size	104	172
Age at diagnosis, mean (SD)	56.9 (9.1)	56.1 (9.0)
Male Gender	78 (75.0%)	154 (89.5%)
Race		
Caucasian	59 (56.7%)	146 (84.9%)
African American	44 (42.3%)	26 (15.1%)
Smoking status		
Never	7 (6.7%)	43 (25.0%)
Current smoker	54 (51.9%)	51 (29.7%)
Quit smoking	40 (38.5%)	73 (42.4%)
Overall stage		
1	4 (3.8%)	4 (2.3%)
2	12 (11.5%)	6 (3.5%)
3	11 (10.6%)	26 (15.1%)
4a	62 (59.6%)	113 (65.7%)
4b	5 (4.8%)	20 (11.6%)
4c	9 (8.7%)	2 (1.2%)
Smoking, pack(-)years *	30 (15, 40)	20 (0, 40)
Chemotherapeutic agent		
None	22 (21.2%)	32 (18.6%)
Cisplatin or carbo/taxol	34 (32.7%)	57 (33.1%)
Cetuximab	13 (12.5%)	18 (10.5%)
Outcomes available		
Recurrence	29 (27.9%)	29 (16.9%)
Persistence	27 (26.0%)	30 (17.4%)
Local recurrence	39 (37.5%)	26 (15.1%)
Regional metastasis	27 (26.0%)	37 (21.5%)
Distant metastasis	10 (9.6%)	14 (8.1%)
Mortality	52 (50.0%)	43 (25.0%)
Recurrence free survival *	21 (10, 44)	28 (9, 61)

* Continuous variables that are skewed given as median, IQR (Inter-quartile range). Some cells do not add to 100% of the sample size because of missing or non-relevant data.

**Table 2:** Patient and tumor characteristics by treatment group

	Cisplatin & carbotaxol	Cetuximab	Unknown chemotherapy	P value
**Size of treatment group**	97	34	77	
HPV serostatus *				
HPV (-)	34 (35%)	13 (38%)	20 (26%)	0.86
HPV (+)	57 (59%)	18 (53%)	42 (55%)	
Overall stage *				
1	0 (0%)	0 (0%)	1 (1%)	<0.05
2	1 (1%)	1 (3%)	2 (3%)	
3	11 (11%)	1 (3%)	11 (14%)	
4a	68 (70%)	26 (76%)	47 (61%)	
4b	11 (11%)	4 (12%)	11 (14%)	
4c	6 (6%)	1 (3%)	1 (1%)	
**Primary tumor site**				
Tonsil	61 (63%)	20 (59%)	50 (65%)	0.19
BOT	34 (35%)	13 (38%)	27 (35%)	
Oropharynx Wall/Soft Palate	2 (2%)	1 (3%)	0 (0%)	
Heavy vs. light smoking (<10 vs. 10+ pack-years) *				
Never	14 (14%)	6 (18%)	18 (23%)	0.41
<10 pack-yrs.	7 (7%)	2 (6%)	3 (4%)	
10+ pack-yrs.	73 (75%)	26 (76%)	56 (73%)	
Current smoker *				
Never	14 (14%)	6 (18%)	18 (23%)	0.45
Current smoker	38 (39%)	13 (38%)	27 (35%)	
Quit smoking	42 (43%)	14 (41%)	31 (40%)	
**Cum. smoking exposure (pack-years), median (IQR)**	30.0 (10.0, 40.0)	30.0 (10.0, 50.0)	24.0 (3.0, 40.0)	0.58
History of alcohol abuse *				
No History	59 (61%)	18 (53%)	40 (52%)	0.80
Positive History	37 (38%)	16 (47%)	36 (47%)	
Age at diagnosis, median (IQR) *	54.0 (49.0, 60.0)	60.0 (56.0, 68.0)	56.0 (50.0, 65.0)	<0.05
**Gender**				
Male	84 (87%)	28 (82%)	63 (82%)	0.76
Female	13 (13%)	6 (18%)	14 (18%)	
Race or ethnicity *				
White	65 (67%)	23 (68%)	62 (81%)	0.23
African American	32 (33%)	11 (32%)	14 (18%)	
Recurrence Free survival, median (IQR) *	27.5 (8.0, 63.0)	16.0 (0.0, 41.0)	21.8 (6.0, 54.5)	0.15
**Total survival, median (IQR)**	32.0 (13.0, 63.0)	20.0 (11.0, 42.0)	25.0 (16.0, 55.0)	0.27
**Mortality rate**	38 (39%)	13 (38%)	25 (32%)	0.37
**Recurrence rate**	17 (18%)	8 (24%)	16 (21%)	0.70
**Persistence rate**	17 (18%)	12 (35%)	13 (17%)	0.15

* Variables may not add to 100% due to missing data.

### Survival

**Figure 1:**
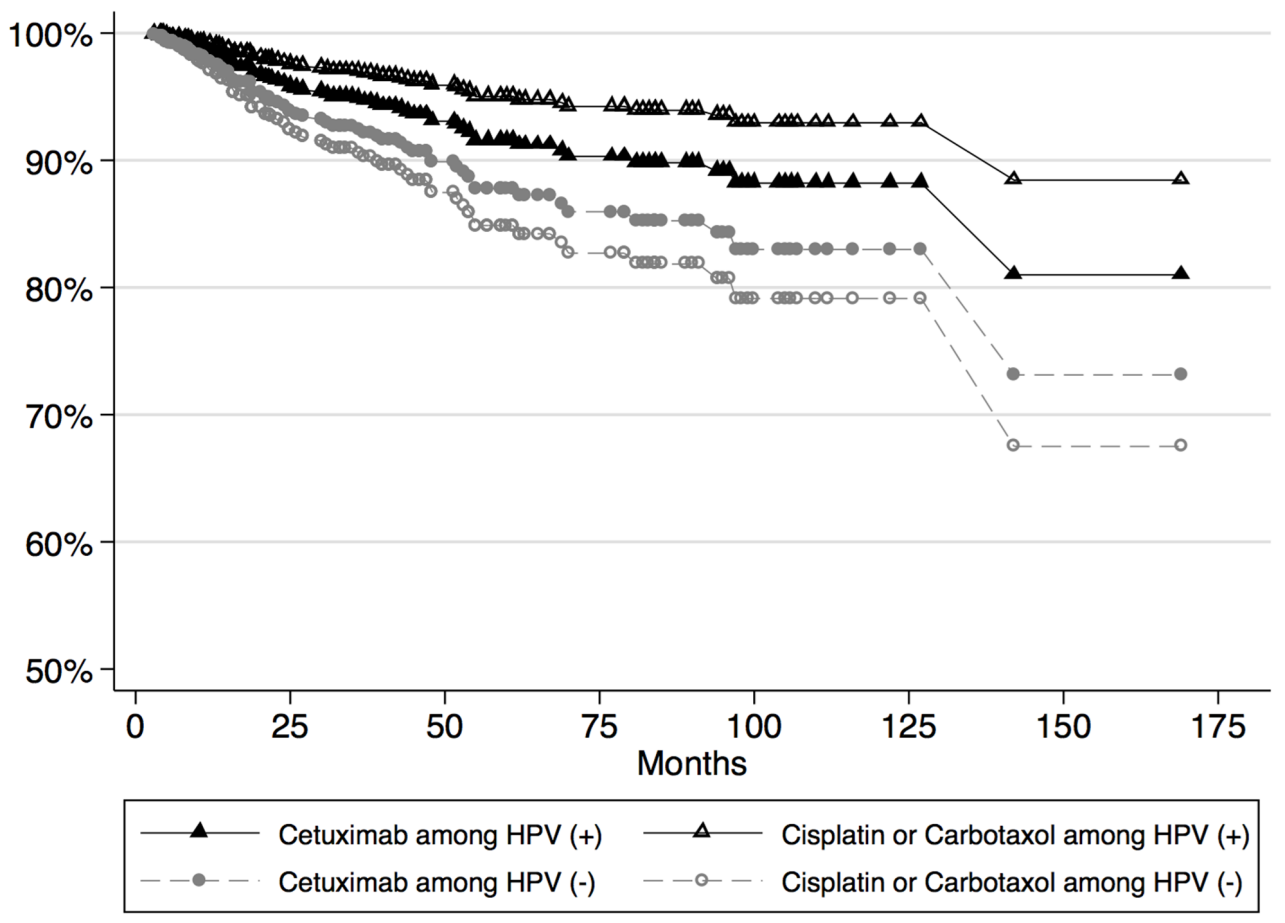
Survival plots for patients based on chemotherapy received and HPV status

**Figure 2:**
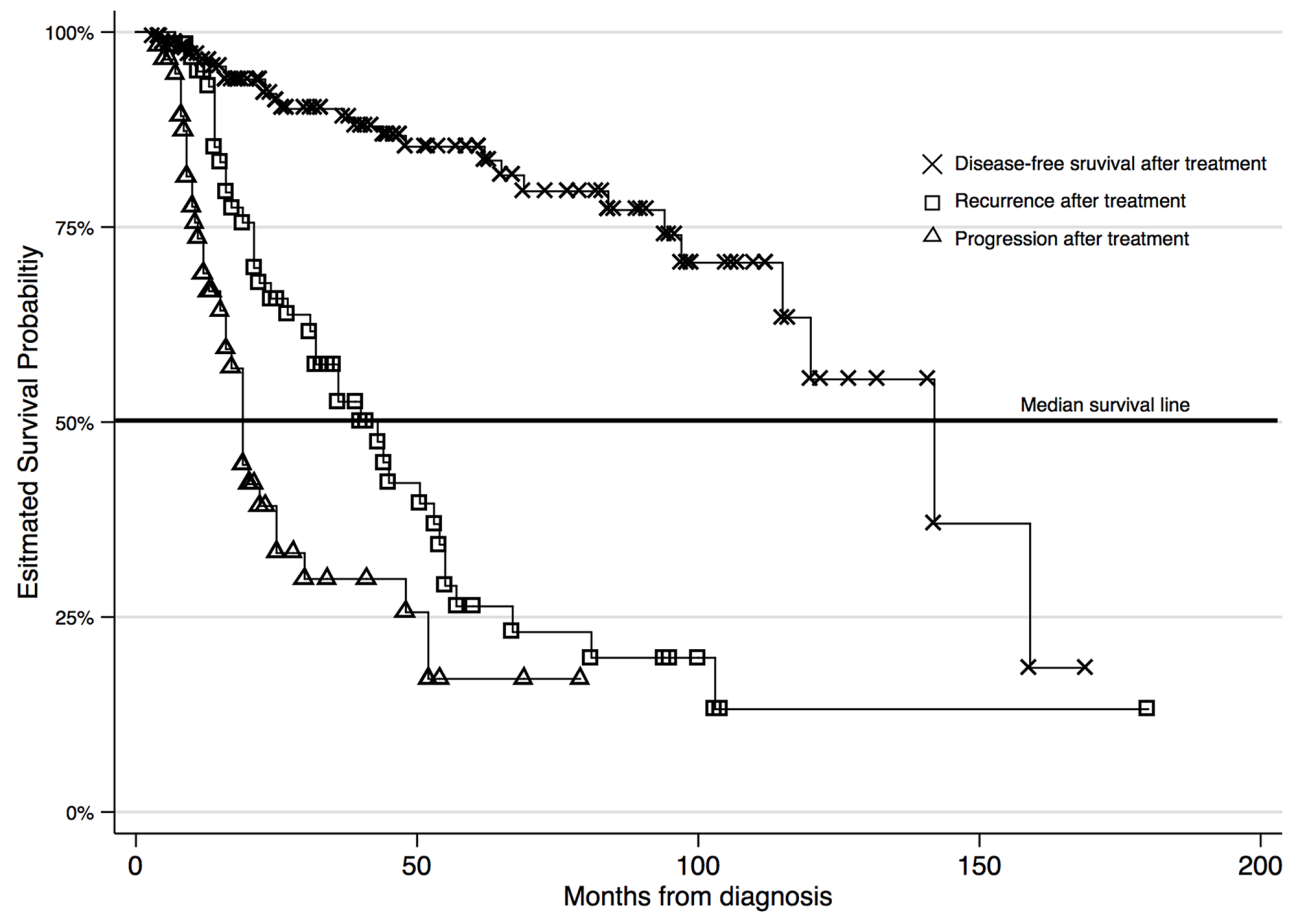
Survival for HPV positive OPSCC patients only based on disease status

A total of 304 participants were followed over an average survival period of 38.8±34.9 months. The length of survival ranged from 3 to 180 months. At 15 years of follow up, the study was administratively censored. With regards to mortality, the unadjusted 15-year period prevalence was 37.7%. The incidence rate estimated over 10897.5 person-years at risk was 9.7 lives lost per 1000 person years. The survival functions of OPSCC patients based on their HPV status and chemotherapy used as a radiosensitizer is shown in Figure 1 and survival by recurrence in HPV + patients only is shown in Figure 2.

Recurrent (RD) and persistent (PD) disease was more prevalent in HPV(-) patients (RD=27.9%, PD=26.0%) as expected; however these outcomes were observed in a substantial number of HPV(+) positive patients as well (RD=16.9%, PD=17.4%)*.* Local recurrence, regional and distant metastases were also more prevalent in HPV(-) patients (LR=37.5%, RM=26.0%, DM=9.6%) compared to the HPV(+) patients (LR=15.1%, RM=21.5%, DM=8.1%). The crude mortality rate was significantly higher for HPV(-) patients (50%) compared to HPV(+) patients (25%) and the difference was statistically significant (P<0.001). HPV (+) patients had longer recurrence-free survival (RFS) compared to HPV(-) patients (median RFS is 21 months for HPV(-) vs. 28 months for HPV(+); P<0.001).

After, adjustment for age, sex, and stage at presentation the hazard ratio for HPV(+) patients vs. HPV(-) patients was 0.30 [95% CI = 0.20, 0.41]. The difference in hazard corresponded to a 3.16 times longer survival for HPV (+) patients [95% CI = 2.81, 3.55] compared to HPV(-) patients. The majority of participants (N=221 or 72.7%) received upfront chemotherapy as a radiosensitizer, while 23 participants (7.6%) received adjuvant chemotherapy. The remaining 57 patients (18.7%) did not receive chemotherapy, but had radiation alone or surgery (+/-) RT. When looking at the chemotherapeutics utilized, 32.0% were treated with either platinum based therapy, 11.2% received cetuximab only, and 13.2% patients received cisplatin followed by cetuximab due to complications of treatment. The remaining patients that were treated with chemotherapy could not have the exact therapeutic regimen identified and were therefore excluded from further analysis.

### Survival analysis by radiosensitizing chemotherapy regimen

The hazard rate for patients receiving cisplatin or carboplatin/paclitaxel was set as the reference group for all chemotherapy regimens. In univariate analysis, patients taking cetuximab only had a higher hazard of death compared to the reference group (HR=1.18 [95% CI = 1.06, 1.32]) indicating a favorable survival profile for platinum-based therapy. Patients who originally took cisplatin and were subsequently treated with cetuximab had a univariate HR=0.31 [95% CI = 0.27, 0.39]. In univariate analysis, cetuximab was associated with a 25% decrease in relapse free survival [95% CI =14%, 33%]. In the multivariable analysis the hazard ratio for cetuximab compared to cisplatin or carboplatin/paclitaxel was HR=0.77 [95% CI = 0.67, 0.90] in the HPV - group, suggesting more favorable outcomes for the patients on cetuximab in this group. However, in the HPV + cohort, the hazard ratio was 1.88 [95% CI = 1.42, 2.50] for those patients treated with cetuximab vs platinum-based therapy.

## DISCUSSION

Our data suggested that cetuximab as a radiosensitizing agent demonstrates inferior efficacy compared to platinum based chemotherapeutics in HPV + OPSCC. The cetuximab only cohort demonstrated almost twice the mortality when compared to those treated with platinum-based therapy (HR = 1.88 [95% CI = 1.42, 2.50]). These results are similar to those seen in other studies. Koutcher et al published a retrospective review of 174 patients with locally-advanced head and neck cancer patients treated with radiotherapy and cetuximab or cisplatin.[8] The data showed a 2-year locoregional failure (LRF) rate of 5.7% vs. 39.9% in favor of cisplatin (p = < 0.0001). The 2-year failure free survival (FFS) was drastically superior in the cisplatin group: 87.4% vs. 44.5% (p < 0.0001). When multivariate analysis (MVA) was used to address prognostic imbalances, treatment with cisplatin showed HRs of 0.09, 0.18, and 0.32 for LRF, FFS, and overall survival, respectively, when compared to cetuximab. Late Grade 3 or 4 toxicity was similar in the two groups occurring in 21 of 125 patients (16.8%) in the cisplatin group and in 10 of 46 patients (21.7%) in the cetuximab group (p = 0.46). While this study included patients with laryngeal, hypopharyngeal and oropharyngeal carcinoma and did not investigate the HPV status, the majority of the patients (≥70%) in each arm had oropharyngeal carcinoma and a fair proportion were non-smokers (34% and 39% in the cisplatin and cetuximab groups, respectively). Ou et al compared 265 patients who had been treated with cisplatin vs cetuximab with concurrent radiation with a subset of 88 patients that had OPSCC with known p16 status.[14] They found that in the entire population, the 5-year progression free survival and locoregional control (LRC) were 51.7% vs. 36.9% (p = 0.01) and 74.2% vs. 51.2% (p = 0.002), both in favor of platinum-based therapy. When looking at the p16-positive subgroup, 5-year LRC rates was significantly better in the cisplatin group compared to cetuximab group (97.4% vs. 71.4%; p = 0.01). However, 5-year OS, DSS and disease control of the p16+ subgroup was not significantly different between the two treatment groups. Additionally, two retrospective studies have demonstrated no difference in survival outcomes for p16+ patients treated with platinum-based therapy versus cetuximab.[15, 16] However, within those studies, the non-smoking rates among their patients were approximately 50% in each arm as compared to the >70% seen in our study and the two aforementioned manuscripts. This may explain the disparity seen in these outcomes due to the higher survival rates and disease responsiveness seen in the HPV + non-smoking population as opposed to the “intermediate risk” patients described in the RTOG study (HPV positive with >10 pack year smoking history).

Biologically, the result of decreased tumor sensitivity to cetuximab is plausible since an inverse relationship has been demonstrated between HPV status and EGFR expression. Hu et al[11] showed in 208 OPSCC patient tissue samples (138 p16+ and 70 p16-) that p16+ was associated with approximately one-third the immunohistochemistry (IHC) staining for EGFR in the cell membrane as p16- tumors (p<.001). Low-levels of EGFR membranous expression was associated with improved OS and DFS in the entire cohort (p=.001 and p<.001, respectively) and within the p-16+ patients only (p=.0248 and p=.002, respectively). Rhie et al[12] compared genomic copy number variations in 58 p16+ and – OPSCC patients. They found a gain in copy number in the p16- patients only. Nakano et al[13] confirmed the results of the studies seen above. They analyzed the presence of high-risk HPV using in situ hybridization (ISH), protein expressions of p16 and EGFR using IHC, and the EGFR gene copy number gain using chromogenic in situ hybridization (CISH) in 105 cases of OPSCC. EGFR gene copy number gain was detected in 12.4% of the OPSCCs and was correlated with EGFR protein overexpression (P =.0667) and worse overall survival (P<.0001). HPV infection and EGFR gene copy number gain (EGFR CISH positive) were mutually exclusive with none of the p16+ patient samples showing EGFR amplification. EGFR protein overexpression was significantly associated with a positive history of smoking (p=.0112), which may explain why our data showed an improved response in our HPV negative patients treated with cetuximab. The HPV-negative/EGFR CISH–positive OPSCCs had significantly worse overall survival than did the HPV-positive/EGFR CISH–negative OPSCCs and HPV-negative/EGFR CISH–negative OPSCCs (P <.0001 and P <.0001, respectively). The EGFR CISH–negative OPSCCs had a favorable prognosis irrespective of HPV infection.

Decreased toxicity has been the guiding rationale for the incorporation of cetuximab in head and neck cancer therapy. However, several of the studies with direct comparisons between the platinum-based chemotherapy and cetuximab have not demonstrated these findings. Walsh et al.[17] showed in a single retrospective review of 48 patients that the cetuximab group experienced significantly higher prevalence of toxicity – grade ≥3 oral mucositis (p = 0.014), skin dermatitis (p = 0.0004), P10% weight loss (p = 0.03), and enteral feeding requirement (p = 0.05). In a randomized phase II trial that was published in 2015 and had to be stopped early because of poor accrual, the investigators noted that although there were more hematologic, renal, and GI toxicities in the platinum-based arm, cutaneous toxicity and the need for nutritional support was more frequent in the cetuximab arm. Additionally, serious adverse events related to treatment, including four versus one toxic deaths, were higher in the cetuximab arm (19%v 3%, P =.044).[18] Koutcher et al[8] did not find any differences in the treatment arms in late toxicity or feeding tube dependence in their series of 174 patients treated with either cetuximab or cisplatin. Finally, in the series from MD Anderson of 300 HPV positive patients, the patients treated with platin agents had a greater incidence of grade 3 anemia, neutropenia, and thrombocytopenia compared to cetuximab (p <.001), but the cetuximab patients had a 19% higher rate of grade 3 mucositis (88% vs 69%) than those treated with cisplatin.[15] While the toxicity profile may not be better for the traditional, platinum-based therapy, the side effect profile of cetuximab is worth noting.

The results of our data contain some limitations. The retrospective nature of our study carries with it the usual limitations including selection bias for treatment. Certainly, the cetuximab patients could have been chosen based on increased morbidity, which may account for some of the survival differences seen in our population. Additionally, many of our patients were treated at different sites thus making it difficult to account for the heterogeneity in radiation and chemotherapy delivery. We also did not have detailed data on weekly versus three-dose cisplatin, nor on treatment compliance or the toxicity profile of the treatment arms. Lastly, there was a slightly higher percentage of stage 4 disease in the cetuximab only population compared to the cisplatin cohort (4%), but this should have been accounted for within the multivariable analysis.

Our data suggest that cetuximab may have inferior outcomes in HPV-associated OPSCC compared to traditional HPV-negative HNSCC. From a mechanistic standpoint, these results are plausible given the inverse relationship between HPV seropositivity and EGFR expression. Due to the improved prognosis and sensitivity to treatment of HPV positive OPSCC, the use of cetuximab has been postulated as a possible way of de-escalating therapy to eventually decrease the morbidity. The retrospective nature of this study make our observations descriptive and hypothesis generating. However, until the results of an adequately powered, prospective randomized controlled trial comparing the two treatments are released, the use of cetuximab in HPV –associated OPSCC should be undertaken with these data in mind.

## MATERIALS AND METHODS

After approval from the LSU Health Shreveport institutional review board, a single institutional retrospective dataset analysis was conducted. The inclusion criteria were defined as adult patients with OPSCC and known p16 and/or HPV status. For some patients, retrospective p16 testing was performed since it was only being conducted on a routine basis for the past 7 years. Exclusion criteria were if concurrent malignancies or metastatic disease were present at the time of diagnosis, if there was a previously treated malignancy of the head and neck, or if the patient had previous irradiation to the head or neck. Medical records were reviewed to obtain the patients’ demographic, clinical, therapeutic, radiologic, and pathologic data. Patients were classified as current smokers if actively smoking within a 6-month period before diagnosis, former smokers, or never-smokers. Tumor staging was according to the American Joint Committee on Cancer 2007 staging system. The overall treatment strategy for patients was determined by presentation at a weekly multidisciplinary conference, but the ultimate decision of what chemotherapeutic agent to administer was left to the treating medical oncologist.

### Statistical analysis

The primary outcomes of interest were 1) overall survival and 2) relapse free survival. Pearson Chi-square tests were used to compare proportions between subgroups. One-way analysis of variance was used to compare the continuous variable age between subgroups. Kaplan–Meier method was used to produce survival curves, and comparisons between survival curves were made using the log-rank test. The survival functions comparing subgroups of chemotherapy were analyzed using semi-parametric (i.e. Cox proportional hazards models) and fully parametric regression with Weibull distributions. Multivariable models were adjusted for age at diagnosis, gender, race, chemotherapy, radiotherapy, and cancer stage. We estimated hazard ratios (HR) and 95% Confidence Intervals [95% CI] based on the survival functions of two or more subgroups with different person months at risk of death.

## SUPPLEMENTARY MATERIALS TABLE



## Abbreviations

(HPV): Human papillomavirus
(OPSCC): oropharyngeal squamous cell carcinoma
(EGFR): epidermal growth factor receptor
(HR): hazard ratios
(95% CI): 95% Confidence Intervals
(RD): Recurrent disease
(PD): persistent disease
(RFS): recurrence free survival
(CISH): chromogenic in situ hybridization
(LRC): locoregional control
(MVA): multivariate analysis
(LRF): locoregional failure
(FFS): failure free survival
