# Supplementary material for: Survival outcomes based on systemic agent used concurrently with radiation in human-papillomavirus associated oropharyngeal cancer
Source: Oncotarget. 2017 Aug 10;8(41):70907–15. doi: 10.18632/oncotarget.20197 (PMC5642607; doi:10.18632/oncotarget.20197)
Supplement: Supplementary file 1 [file oncotarget-08-70907-s001.pdf]

Survival outcomes based on systemic agent used concurrently with radiation in human-papillomavirus associated oropharyngeal cancer

SUPPLEMENTARY MATERIALS

Supplementary Table 1: Outcomes among HPV-positive smokers

	Cisplatin or carbotaxol	Cetuximab	Unknown chemotherapy	p-value
**Primary tumor site**				0.76
Tonsil	13 (68%)	4 (80%)	5 (62%)	
BOT	6 (32%)	1 (20%)	3 (38%)	
**Recurrence**	2 (11%)	2 (40%)	0 (0%)	0.21
**Persistence**	3 (16%)	2 (40%)	2 (25%)	0.48
**Regional metastasis**	4 (21%)	2 (40%)	2 (25%)	0.68
**Distant metastasis**	1 (5%)	0 (0%)	0 (0%)	0.70
**Death**	8 (42%)	2 (40%)	1 (12%)	0.33
**Survival time in years, median (IQR)**	2.1 (1.2, 6.4)	1.1 (1.0, 1.3)	1.8 (0.8, 2.8)	0.36
**RFS in months, median (IQR)**	25.0 (12.0, 77.0)	14.5 (12.5, 31.0)	15.8 (9.0, 33.0)	0.67
